# I2I - From illusion to illumination: A neoteric deep learning model for recognizing medical situation actions using depth sensors data

**DOI:** 10.1371/journal.pone.0337646

**Published:** 2026-05-18

**Authors:** Saima Sultana, Eraj Tanweer, Muhammad Mansoor Alam, Sadia Nazim, Mukesh Prasad, Jawahir Che Mustapha, Mazliham Mohd Su’ud

**Affiliations:** 1 College of Computer Science and Information Systems, Institute of Business Management, Karachi, Sindh, Pakistan; 2 Malaysian Institute of Information Technology, Universiti Kuala Lumpur, Jalan Sultan Ismail, Kuala Lumpur, Malaysia; 3 Software Engineering Department, NED University of Engineering & Technology, University Road, Karachi, Pakistan; 4 Faculty of Computing, Riphah International University, Islamabad, Pakistan; 5 Department of Computer Science, Bahria University, Islamabad, Pakistan; 6 Faculty of Engineering and Information technology, School of Computer Science, University of Technology Sydney, Sydney, Australia; 7 Faculty of Computing and Informatics, Multimedia University, Cyberjaya, Malaysia; University of Portsmouth, UNITED KINGDOM OF GREAT BRITAIN AND NORTHERN IRELAND

## Abstract

Among many issues, Robot vision experiences illumination challenges very frequently. Existing Human Action Recognition techniques perform excellently in state of the art, instead of scarce consideration on the vital issue of illumination. The illumination concern becomes highly sensitive when the Robot observes a medical-related action. The illumination severely affects the correct recognition of the action. Resultantly, misclassification of a medical action may lead to irreparable loss. To gauge the sensitivity of the concern, the current study proposes a deep learning-based model I2I (Illusion to Illumination). The model effectually identifies medical actions even in dark environments with sufficient accuracy. I2I model depth data has been selected from the NTU RGB+D dataset to judge the efficacy. The features are extracted from depth data using the Histogram of Depth (HoD) and provided to the I2I model to recognize actions. A threshold mechanism is applied to select depth data’s most prominent and valuable features. The efficacy and superiority of the I2I model are proven by comparing its performance with state-of-the-art research and provides **91.15%** recognition accuracy.

## 1. Introduction

Robots are good co-workers of humans and perform difficult tasks through the visual processing capabilities of Robot vision. During the visual processing, some of the misleading issues become error-prone like clutter scenes, occlusion, inappropriate visual input, and illumination. Many researchers have considered numerous problems with the lighting condition consideration as constant and take it as not affecting the recognition [1]. In order to deal with appropriate/insufficient lighting conditions, there is always a high probability of misrecognition of an action performed by humans. The illumination issue becomes more sensitive when the recognition is related to health conditions [2]. Misrecognition could lead to irreparable loss. The situation becomes life-threatening. So, it is essential to pay more prioritized attention to finding a more accurate solution for the illumination issue [3].

**Fig 1:**
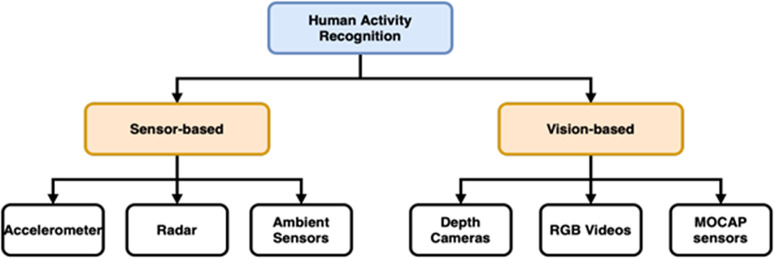
A comprehensive HAR method hierarchy. HAR is usually divided between techniques based on sensors and approaches based on vision. The two main division separation is subject to the nature of sensors, where each sensor data is usable by multiple domains.

The researchers usually consider Human Action recognition data in RGB video, depth, infrared, or skeleton format [4]. To deal with the illumination issue, it could be improved for recognition accuracy using Human action recognition data in Depth image format. The depth data could be easily gathered using Kinect V2 cameras. Fig 1 describes it well.

Technically endorsing, the skeleton, RGB image, point cloud, Radar, and RGB image may be used to categorize data gathering modalities, instead, HAR can be classified as variants of data as seen in Fig 1. Due to its ease of operation and availability, RGB picture is often the most used type of data, offering a multitude of information. The human body’s posture may be precisely described by the skeleton, which also provides high-level performance in settings with few objects by encoding with body joints. In spatial modeling, autonomous driving, and robot navigation, point-cloud, which is recorded by a depth-sensing camera and may offer 3D information such as depth and distance, is a crucial technological advancement. It is less typical to employ radar, as it is capable of realizing through-wall identification, and it is impervious to outside influences like noise and shifts in lighting [5].

### 1.1. Depth data for medical action recognition

Depth cameras generate distinct streams or distinct depictions of an identical activity. Certain studies have tried to combine streams of available data in different formats to enhance categorization. One may argue that the best methods for differentiating actions with wide motions are those based on skeletons. Skeletons-based models, however, are not as effective for tasks involving considering illumination issues [6].

In addition to sensor-based systems, depth maps and video camera inputs have been used to identify medical conditions in a variety of situations, including fall detection, depression detection, senior assistance, and more [7,8]. These methods are far less intrusive and might be scaled, even if they are not as precise as sensors affixed to the body. They need video cameras, depth sensors, and gadgets like Kinect for equipment. A few of these systems use RGB data as features directly at the frame level. In contrast, some extract the features from depth data with posture estimation methods [9] and then apply it to determine medical issues.

The practice of employing sensors and algorithms to recognize and categorize human behaviors based on the data gathered is known as Human activity recognition. The purpose of HAR is to make it possible for machines, such as computers or robots, to comprehend and interpret human behavior in a manner that is comparable to what and how humans do so [10].

**Fig 2:**
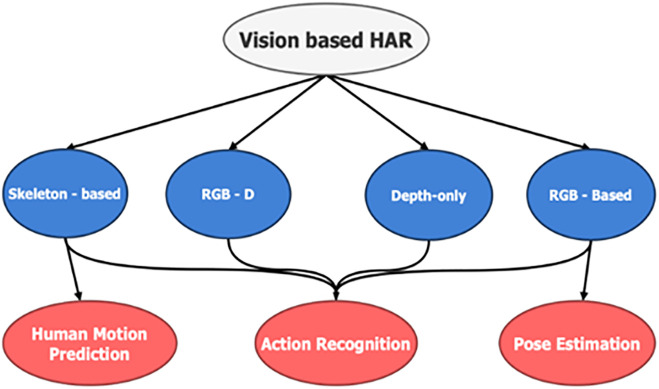
The data modalities that are frequently employed in vision-based HAR techniques. Each modality has multiple uses, thus vision-based approaches further classification might take place and associated for tasks that follow. The most noteworthy, but not limited to the ones are posture estimation, action identification, and human motion prediction.

Since depth sequences provide geometric information and are immune to variations in light, they are helpful for action recognition. Due to their high computational costs and requirement for massive amounts of training data, many existing action recognition techniques are constrained [11]. Depth data streams are available as 2D images in various datasets and have been thoroughly investigated by researchers. Contour extraction of different subjects based on depth information can be used to detect humans when the input is a stereoscopic picture or video [12]. Fig 2 describes the phenomena well.

### 1.2. Depth data for illumination problem

For enhanced performance in illumination issue resolution, numerous techniques are applied by researchers. Resultantly it is revealed that Depth data is not sensitive to illumination and could be collected using a Kinect II sensor. The collected data is processed to combine the benefits of deep learning (DL) models [13]. These models usually entail taking the data and extracting viable and prominent features, which are then fed into a deep learning model for categorization [14].

The great heterogeneity in human activities and the variety of data sources that may be used to identify them is one of the main challenges in HAR. To address these problems, researchers have developed several methods for feature extraction, feature selection, and classification. Feature extraction techniques aim to extract relevant information from the data [15]. Feature selection strategies choose a subset of the most pertinent qualities from the data. Classification approaches, on the other hand, prefer to utilize a neural network model that can correctly predict the activity label based on the characteristics [16].

The identification of human activity using depth data can be difficult because a variety of illumination characteristics might impact image quality and in turn, the precision of activity recognition. For example, low illumination might make it challenging to discern between several tasks. Previous studies have identified this problem using thermal cameras [17].

While the NTU RGB+D dataset itself does not explicitly capture sequences under varying illuminations, the contribution of illumination variability in this work is done by simulating such through data preprocessing and depth-based analysis. Because depth data intrinsically captures structural geometry rather than color intensity, this makes it a natural baseline for illumination invariance. Accordingly, the proposed I2I framework is designed to test the network’s resilience to synthetic brightness and contrast variations, reflecting practical illumination shifts that may happen in real robotic environments. Future extensions will consider the use of datasets that have controlled illumination diversity, enabling empirical validation of the robustness observed here. In this respect, the contribution regarding illumination has been maintained by showing that the proposed framework works effectively not only under depth-based robustness but also under changing light conditions and thus provides reliable recognition in complex visual settings.

The current study proposes a novel Deep Learning based model, I2I for categorizing Medical actions of human behaviors observed by a Robot, to address illumination issues. The current study selects all available action data to extract meaningful and important features from the provided depth data. Following that, the important features from medical action performed by the subject would be processed to provide for the classification of the novel neural network. The Neural Network I2I makes use of those features to recognize human behaviors.

For this experimental study, the widely used dataset NTU RGB+D [18] has been chosen and evaluated with high accuracy, precision, recall, and f1-score parameters. The suggested method classifies the medical actions on depth images better than state-of-the-art approaches [19].

The primary significance of this research can be condensed into the following key points:

- A deep Learning-based novel algorithm I2I, has been proposed for Robot vision to recognize and classify health-hazardous actions in illumination-affected scenarios.
- To assess the performance of the novel proposed algorithm, depth data from the NTU RGB+D dataset have been selected. Which utilizes all available related depth data to generate features.
- Histogram of Depth (HoD) has been utilized to generate features from Depth data.
- The strongest features have been selected for the empirical study and assisted critically for their performance.
- By implementing the I2I model technique, an extensive and exhaustive empirical study resemblance with state of the art is performed to judge the impact of illumination on the identification of health-related actions by Robot vision.

Notably, there hasn’t been any research that we are aware of whereby depth image data are processed by a deep neural network model. Finding a research that employs depth through HoD to deep Neural network models is even less common for action recognition; when the architecture’s full potential is utilized.

The rest of the paper is organized into 8 sections. Section 1 comprises of introduction to the research area. Furthermore Section 2 highlights related state-of-the-art research work. Section 3 explains the Methodology, including dataset, feature selection, and network architecture. Section 4 explains the experimental setup and implementation details of the research. The evaluation metric and evaluation protocol results are explained in Sections 5 and 6 respectively. Afterward, the research is accomplished with a discussion in Section 7 and a conclusion in Section 8.

## 2. Related work

As mentioned in the introduction, early depth-based HAR research included techniques with manually created features and visual pictures captured with common RGB cameras. Deep learning techniques started to be used for videos as well with their simple and complex architectures, driven by their success in image processing tasks [20]. Numerous studies have surfaced to document the developments, approaches, and uses influencing this ever-evolving field. These thorough assessments provide insightful critiques on the development of HAR made possible by deep learning and machine learning methods.

The research study [21] utilizes Depth data to assess HAR and proposes a hybrid network ConvLSTM and compares and proposes two methods for real-time raw depth video sequences-based human action recognition (HAR). The convolutional long short-term memory unit, or ConvLSTM, is the foundation of both ideas; however, long-term learning and architecture are different. The proposed model accomplishes 75.26% accuracy for Cross Subject and 75.45% accuracy for Cross view. The research [22] presents a histogram-based new descriptor for activity recognition from videos at MSRDaily dataset acquired by a depth sensor and produces 30.56% accuracy. The research study [23] proposes a framework for human activity recognition from depth video sequences of the MSRDaily 3D action dataset. It is captured by a depth camera using a hyper surface normal in a depth sequence to form the poly normal which is used to characterize the local motion and shape information jointly.

Notably, the research [11] highlights how important it is to improve HAR systems and provides an effective method for depth sequence-based human action recognition. Spatial Laplacian and temporal energy pyramids break down the depth sequences into specific frequency bands. The differences and buried levels are used to extract 4D hyper-surface normal and HOG features, respectively. Maximal pooling is used throughout the temporal segments to reduce outliers and produce a compact representation of a sequence. Additionally, local sparse representations are included to get additional spatial orientation data in nearby communities.

The research [24] proposes an RNN-based Encoder-Decoder framework to predict sequences of 3D atomic motions over time, improving upon existing methods that use single optical flows or 2D trajectories. It explores unsupervised learning across different modalities (RGB, depth, RGB-D) and demonstrates superior performance in activity recognition tasks, outperforming previous state-of-the-art unsupervised methods.

A variety of quick methods have been developed for action recognition using RGB data. The adoption of inexpensive RGB-D sensors, like the Microsoft Kinect, has allowed several researchers to expand their 2D work into 3D. This sensor records 3D skeletal data, RGB (2D) pictures, and depth images [25].

They [26] propose a viable network by transfer learning and the paradigm change towards autonomous feature extraction using deep neural networks, respectively. The research [27] also closes a significant gap in the literature by offering a thorough examination of modern deep learning techniques used with HAR. When taken as a whole, these books not only outline the state of HAR now but also draw attention to its issues and prospects, providing a thorough framework for comprehending its advancement promise within this field. [28,29] Introduces the Greylag Goose Optimization (GGO) algorithm, a swarm-based nature-inspired optimizer modeled on geese migration behavior, demonstrating superior performance and accuracy across UCI datasets, engineering benchmarks, and case studies compared to existing optimization algorithms [30]. The Football Optimization Algorithm (FbOA), inspired by football strategies to balance exploration and exploitation, demonstrates superior performance in solving high-dimensional optimization problems, outperforming state-of-the-art metaheuristic algorithms in speed, accuracy, and robustness.

## 3. Methodology

The field of research on the subject of Human Activity Recognition (HAR) has grown exponentially, producing an immense array of research. Depth image processing is a promising new approach that can handle big corpora with exceptional accuracy in short amounts of time. The methodological approach is explained in this section. To start, the dataset source is described first, emphasizing that our analysis is restricted to the data source. Further specifications include pre-processing and cleaning of data, strong feature selection, processing strategies, and network architecture with design specifications.

### 3.1. Dataset

To find an effective solution for the illumination, the widely used dataset NTU RGB+D has been selected. NTU RGB+D is one of the biggest human activity datasets available that includes infrared, depth maps, RGB films, and 3D skeletons. It has 56880 instances in which one or more people are seen carrying out a certain task. Three Microsoft Kinect II sensors have been used to record videos simultaneously, resulting in multi-view scenarios. RGB films have a resolution of 1920 x 1080 pixels, whereas infrared and depth-map recordings have a resolution of 512 x 424 pixels. For each frame, three-dimensional positions of the 25 major joints in the human body are provided via 3D skeletal data. There are sixty human acts in the dataset among three distinct categories: mutual activities, medical conditions, and daily actions.

The dataset can aid in the advancement of human activity analysis and enable the use of data-hungry techniques like deep learning for this purpose. In the dataset NTU RGB+D all actions are performed by 40 different subjects aged between 10–40 years and each action is captured with 3 different sensors, placed at varying locations of the action subject. As the identification of human action from depth data is not sensitive to lightning conditions and this dataset includes depth data as an array of PNG images computed from each action depth video, it is utilized for resolving illumination issues for Robot vision. It can improve the overall efficiency of the medical action recognition system. The main reason for depth data selection is, that depth information is less affected by lighting conditions as compared to other available formats of recognition information. So definitely would lead to estimating a viable resultant for illumination issues [31].

The Depth data is collected and extracted through the Depth sensor. These sensors provide depth information to process. Smart surveillance, ambient-supported living, human-computer interaction, medical assistive applications, and other applications have made extensive use of human behaviors and activities. The current research areas are in consideration topics in present HAR, which involves implementing both simple and complex solutions. These actions are visually similar, but actually, it is quite different from one another [32].

**Fig 3:**
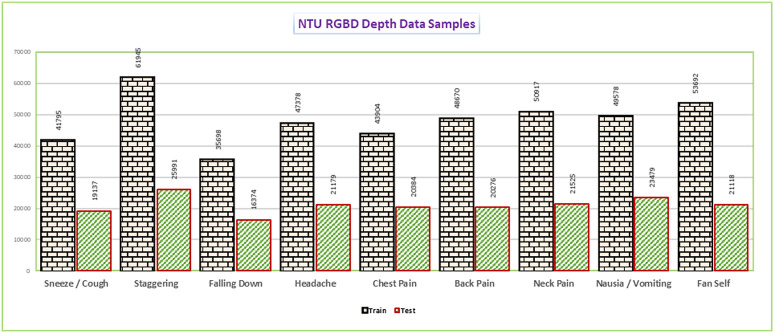
NTU RGB+D depth data distinguished in Train and Test for each action and representing number of samples available separately. The number of samples for each action is unequal in count.

To conduct and evaluate the research study, all medical-related actions available in in NTU RGB+D dataset as 9 distinct medical related action classes have been selected. These classes belong to the medical actions of Sneeze/ Cough, Staggering, Falling Down, Headache, Chest Pain, Back Pain, Neck Pain, Nausea/ Vomiting, and Fan self. Fig 3 provides a one-glance complete data statistics.

### 3.2. Depth data pre-processing and feature selection

Among many available data in varying formats of RGB, depth (RGB-D), Infrared, and skeleton-based data the current study only utilizes a solo format. The study is only interested in finding viable solutions for the illumination issue, so it considers depth data for action recognition of medical conditions [33].

To find viable solutions for illumination issues in Medical action recognition, the current study chose to work with Cross Subject data. In the very first step, the data is arranged in Cross Subject order ranging from Subject 1 to Subject 40 for each action class. Each action sequence is provided in depth data format and is a collection of PNG files, each one comprising 512 x 424 pixels. As to the data suffices enough with no missing values to process by the proposed Deep learning model I2I, so none of the augmentation is performed at data. Depth Feature Extraction Formulation for I2I as Histogram of Depth (HoD).

Later on, the pre-processing of data is to collect important features from depth data. For this purpose, the depth data is resized into a target size of 224 x 224 pixels in a grayscale image, then normalized pixel values to range from 0 to 1. Afterward, the image gradients are computed in the x and y directions to find the magnitude and direction, capturing edge and contour information in the image. The normalized image is split into a grid of cells based on the provided cell size of 16x16. That results in the collection of the gradient magnitudes and directions for each cell. Afterward, a histogram is created of gradient directions (using 9 bins from −180° to 180°) by accumulating magnitudes in each bin. Furthermore, the mean, standard deviation, minimum, and maximum pixel values from the normalized image are calculated and create a combined feature vector by appending the histogram of oriented gradients (HOD) features with the intensity statistics.

### 3.3. The threshold variant feature selection mechanism for I2I model

For the processing of illumination concerns, the current study considers all the depth data samples from the NTU RGB+D dataset. There is a collection of 623,040 depth data samples in the dataset. The current study considers the Histogram of Depth (HoD) to extract features from depth data. The extracted features set comprised 1768 features for each depth sample. These features contribute in current research with the Threshold Variant selection mechanism and compete their resultants with each other to prove the efficacy and significance rationale of the I2I model.

Upon the synthetic observation of extracted features from HoD, it is revealed that those 1768 features contain a large number of 0s in each one. A feature set with a high concentration of zeros indicates that there is little relevant change in a large portion of the retrieved feature space when evaluating depth data. An excess of zero’s in the Histogram of Depth features often represents very little knowledge in the feature set and indicates areas with consistent or little fluctuation in depth, which frequently correlate to backdrop or other areas devoid of distinguishing elements. It proportionally impacts the tasks involved significantly in recognition of sparse representation and consequently, the feature set does not classify the action in an efficient manner.

The discriminative ability required to successfully distinguish actions might be absent from a feature vector that is dominated by zeros. Because many characteristics offer very little information, such a small variety limits the model’s ability to recognize and learn unique patterns. In addition to slowing down training since the model has to sort through inactive features, this sparsity (presence of many zeros) may cause some model parameters that receive a little update to be underutilized. Additionally, the model runs the danger of overfitting due to the small number of active features, possibly latching onto the sparse, non-zero components that would not translate well to fresh data.

Furthermore, this sparsity suggests that dimensionality reduction methods like feature selection or PCA might greatly enhance the feature set’s quality. These methods can improve computation efficiency and the model’s generalization ability by narrowing the feature space to include the most informative elements. There should be some techniques to increase feature variety by adding more descriptors, transformations, or normalizing techniques that can lessen the preponderance of zero’s and produce a more reliable and insightful depiction of depth data.

The idea behind this approach lies in the fact that highly sparse HoD features, with respect to bins that are zero for most samples, do not contribute much to the descriptor’s discriminative power. In depth-based histograms, a bin being zero consistently across most instances indicates that the corresponding spatial-depth pattern hardly occurs in the dataset, hence its limited relevance for class differentiation. Keeping such inactive or rarely activated bins not only increases computational costs but also introduces noise into classifiers, which may degrade generalization. As such, thresholding by zero-value proportion effectively filters redundant or non-informative dimensions while retaining those bins that capture more representative geometric structures.

So, the current study chose to get dimensionality reduction through the threshold method. By implementing a threshold value for several zero’s in each feature, those features were selected for the experimental study.

**Fig 4:**
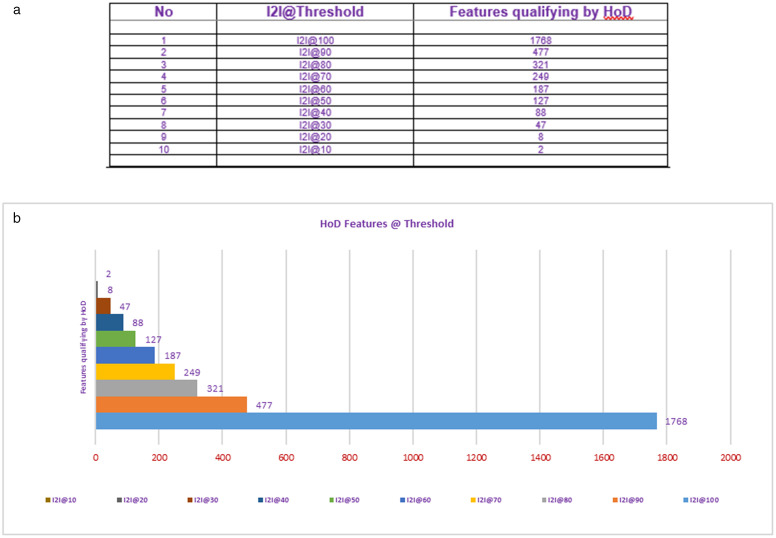
(a) Represents I2I Experimental Threshold Variant Feature Selection Mechanism with detail of number of features qualified in tabular form. While (b) represents the graphical view of number of features qualified by HoD. The minimum number of features qualified by I2I@10 and maximum qualified by I2I@90.

**Fig 5:**
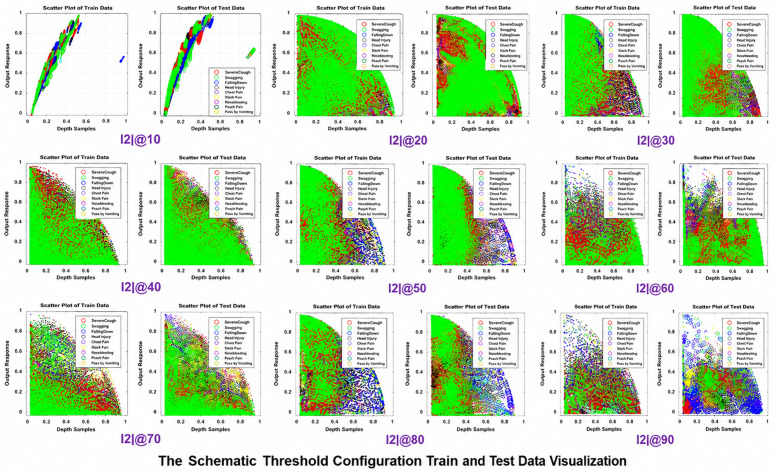
The schematic threshold configuration data visualization for each train and test feature samples. The whole data features qualified by HoD consisting of each action samples. The number of features varies for each scheme and represents the whole corpus range visualization in 2D scatter plot.

The variant schemes of Threshold Configuration are based on selecting number of zero’s in the feature set. The I2I@100 contains all features having zero’s while I2I@90 contains 477 features having 90% of zero’s in their feature set. Similarly, I2I@10 have only 2 features which means among all features only 2 features had 10% zero’s in their feature set. For example, when the threshold is set from total to 50% only 127 features were selected. This proves that selecting the whole feature set might result in overfitting. The number of features qualified for different threshold values is described in Fig 4 (a, b) below. The data is visualizable in Fig 5 below.

### 3.4. The proposed model I2I architecture overview

The network design and computational efficiency have a direct impact on how well actions are recognized. One of the useful tools for creating a neural network to evaluate the performance for recognition is the Deep Network Designer. It is included in Matlab R2022a and used to formulate the proposed network model. The following Fig 5 reveals the model composition along with the configuration.

**Fig 6:**
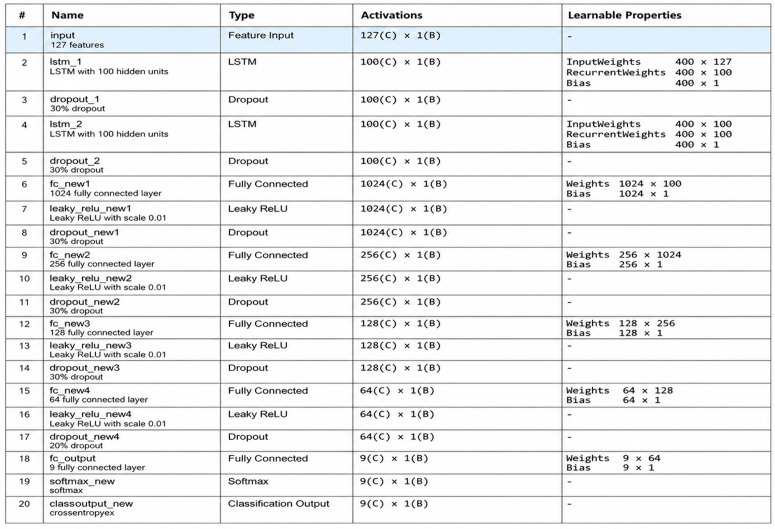
The configuration description of the proposed model I2I. It is comprising of variant layers and its parametric details. From input layer to the classification layer, their types, activations and learnable parameter details are elaborated sufficiently.

**Fig 7:**
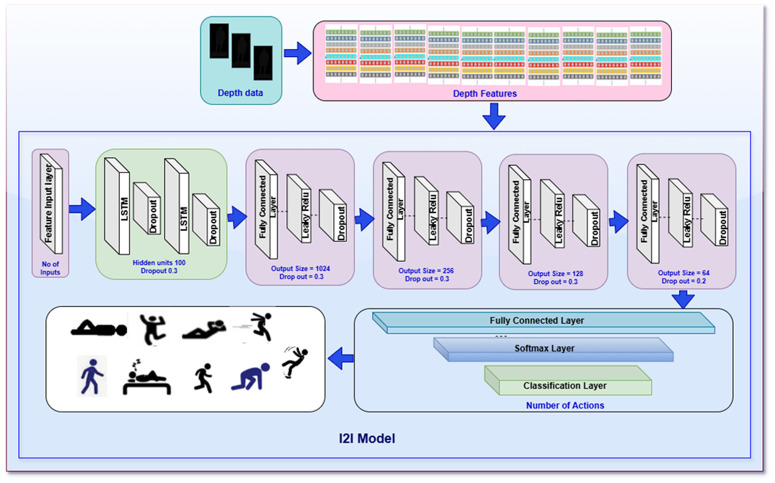
The proposed model I2I specification and its complete system with depth data. The system initiates with depth data images feature input to the first layer of proposed model I2I. The input layer passes the received features to the next two LSTM layers, which is followed by a series of fully connected layers. Those fully connected layers are consisting of Leaky Relu layers which produces final outcomes of the input features to classify the feature in their respective classes.

To recognize medical actions, the proposed network seeks to extract co-occurrence patterns from depth data. Fig 6 exposes the configuration details of the proposed model I2I, while Fig 7 reveals the whole research phenomena well. The thorough explanation of the robustness of depth data-based action recognition using analysis measures reveals challenges. The proposed neural network I2I is expressed as a combination of recurrent neural networks such as LSTM and fully connected layers in various configurations. The present empirical study’s correct categorization is achieved by combining both layers in a way of configuration that outperforms the best of the best for recognition by the actions for Robot vision.

**Fig 8:**
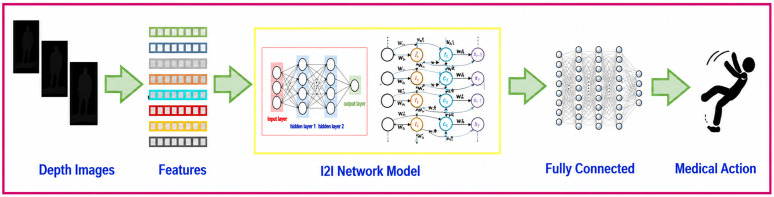
The Depth data recognition with the I2I model in a complete system. The flow of system initiates with depth data images input and features selected with help of HoD. Only a single schematic configuration features are provided to proposed model I2I for classification of medical actions in their respective classes.

First, the feature input layer receives the features in a schematic way of selection with a threshold value. The selected number of features from the schematic threshold configuration is revealed in Fig 8.

Multiple fully connected (FC) layers are used in the I2I model to enable a more thorough and progressive processing of the depth data, which eventually improves the classification capability of the model. From low-level to more intricate, task-specific characteristics, each FC layer first extracts progressively abstract and sophisticated patterns from the input data. In addition to enabling hierarchical information distillation, the successive reduction of layer sizes (for example, from 1024 to 64 neurons) motivates the network to concentrate on the most important patterns while reducing superfluous noise. The model becomes non-linear by adding Leaky ReLU activations after every FC layer. This enables it to identify complex correlations in the data that linear transformations by themselves would miss.

To prevent overfitting and ensure that the model learns generalizable characteristics rather than remembering noise or unimportant information, regular dropout and L2 regularization across layers are also used. By carefully balancing complexity and overfitting management, The I2I model produces a robust model that more effectively captures the patterns required for precise categorization. The overall system diagram is described in Fig 8 as follows,

The sequential data processing capabilities of the neural network architecture I2I take features according to the schematic threshold configuration. 127 features are provided to the I2I network when threshold I2I@50 has been selected and provided for each sample of data to the feature input layer. To identify sequential patterns in the data, the network then makes use of two Long Short-Term Memory (LSTM) layers, each of which has 100 hidden units. A dropout layer randomly removes 30% of connections after each LSTM layer to avoid overfitting. The network comprises a sequence of fully connected layers that gradually reduce the dimensionality from 1024 to 256, 128, and ultimately 64 units after the LSTM layers.

A Leaky ReLU activation function with a modest slope (0.01) follows each fully connected layer, enabling gradient flow even for tiny input values. Each activation is followed by dropout layers with 30% and later 20% rates, which improves generalization even further. A softmax function is used to translate outputs into class probabilities after the last layer, a fully connected layer, maps the processed characteristics to the number of target actions. The network is completed with a classification layer with cross-entropy loss.

The proposed model I2I limitations is crucial for a balanced perspective. The I2I model exhibits sensitivity to noisy data features and outliers of depth images data, which can impact its overall performance and reliability. Acknowledging these challenges, the current study has selected features through HoD and outlined potential mitigation strategies with Schematic Threshold Configuration implementation.

## 4. Experimental setup and implementation details

Using the preprocessing and feature extraction modules covered in Section 3, we first processed all relevant data. For the sake of simplistic observation, the current study has formulated a few thresholds, including I2I@10, I2I@20, I2I@30, I2I@40, I2I@50, I2I@60, I2I@70, I2I@80, and I2I@90 separately. Then, each shuffled data set is split into training and testing sets according to the K fold distribution. K fold Where K = 10 outperforms the I2I model and wins the efficacy with state-of-the-art empirical studies.

The K fold method makes the most use of the data by guaranteeing that every data point is included for both training and testing. As K-fold cross-validation is a widely used technique for training and testing Deep learning models. The method lessens the possibility of overfitting and provides a more accurate indicator of the model’s performance on fresh, untested data. It produces more generic results by dividing the data into several folds and averaging the outcomes, in comparison to a single train-test split. It makes the evaluation more impartial and reliable.

The study’s instruments and technology are mentioned in this section. On a personal workstation running with 16 GB RAM and an Intel CoreTM i7-4600U CPU @ 2.10GHz processor running at 2.70 GHz, all experiments were conducted exhaustively. The empirical study is executed at Matlab R2022a, and the deep architecture has been constructed using Deep Network Designer. We have trained the proposed model using the ‘Adam’ optimizer and trained for 100 epochs with a mini-batch size of 64. The initial learning rate is 0.0001 with a gradient threshold of 0.02.

## 5. Evaluation metric

The standard evaluation metrics are followed to assess the effectiveness of the proposed model. For trustworthy findings, each simulation in this section is cycled through K fold times to determine the Accuracy, Precision, and F1 Scores. The I2I model technique is used to test each extracted feature’s effectiveness in the suggested model by choosing variants of the threshold. The Accuracy, Precision, and F1 scores have been noted and averaged for every observation. These matrices are calculated as follows:


$$\begin{array}{c} \text{Accuracy}=(\text{T P}+\text{T N})/(\text{T P }+\text{ T N }+\text{ F P }+\text{ F N}) \end{array}$$ Eq. (i)

$$\begin{array}{c} \text{Precision}=\text{T P}/\;(\text{T P }+\text{ F P}) \end{array}$$ Eq. (ii)

$$\begin{array}{c} \text{F1Score }=\;\text{2}\text{ x }(\text{P x R})/\;(\text{P }+\text{ R}) \end{array}$$ Eq. (iii)

Where TP, TN, FP, and FN represent the number of True Positive, True Negative, False Positive, and False Negative predictions respectively.

## 6. Evaluation protocol and results

This section conducts an experimental and ablation evaluation study of the proposed methodology using the depth map data of the widely used dataset NTU RGB+D 60. The observational approach finding details to examine the impact of illumination on the recognition approach is revealed in this section. The selection of state-of-the-art empirical studies is subject to similar modality of data from similar dataset and technique to resolve the issue and to perform fair comparative analysis. Table 1 contains all the state-of-the-art research compiled for both Cross subject and Cross view. Table 2 contains the empirical study result evaluation of the Schematic Threshold Configuration method.

**Table 1:** The I2I model performance comparison with the state-of-the-art research exploiting similar studies of Depth image data for action recognition. Each study has performed the evaluation on similar set of data using their peculiar technique. Whilst, the proposed I2I model out performs better than state-of-the-art techniques.

State-of-the-Art Empirical Study	CS	CV
[11] SLTEP(2017)	58.22%	---------
[24] Unsupervised ConvLSTM (2017)	66.20%	---------
[34] Dynamic images (HRP) (2018)	87.08%	84.22%
[35] HDDPDI (2019)	82.43%	87.56%
[36] Multi-view dynamic images (2019)	84.60%	87.30%
[37] Hybrid Descriptors (2021)	88.12	---------
[21] ConvLSTM-Stateless (2023)	75.26%	75.45%
[21] ConvLSTM-Stateful (2023)	80.43%	79.91%
I2I Model	**91.15**	---------

**Table 2:** The I2I model performance at various Schematic Threshold Configurations in terms of Accuracy, Precision, Recall and F1 Score. Each scheme’s performance is captured in the table to compare the performance with each other.

	Accuracy	Precision	Recall	F1 Score
*I2I@10*	21.68	22.76	21.76	21.90
*I2I@20*	30.31	31.13	31.12	31.09
*I2I@30*	63.30	62.90	63.30	62.11
*I2I@40*	72.68	74.01	74.15	73.82
*I2I@50*	78.16	78.22	78.16	77.92
*I2I@60*	82.19	82.35	81.89	82.99
*I2I@70*	84.35	84.76	84.35	83.89
*I2I@80*	86.91	86.78	86.91	85.98
*I2I@90*	**91.15**	**91.28**	**91.32**	**91.26**

Using a single RGB camera with OpenPose and 3D-baseline for skeleton extraction, [11] suggests a real-time human action identification technique that achieves 58.2% accuracy on NTU RGB+D at 15 frames per second using a mobile robot platform powered by an NVIDIA Jetson Xavier [24]. Presents an unsupervised ConvLSTM, uses a recurrent encoder-decoder framework to capture strong spatiotemporal relationships for activity classification across RGB, depth, and RGB-D modalities. In order to achieve state-of-the-art results on huge datasets using only the depth modality, [34] suggests three compact depth-based representations (DDI, DDNI, and DDMNI) as well as a ConvNet-based technique for action recognition. [35] and [36] Introduces a hierarchical dynamic depth projected difference images representation and multi-view CNN framework to effectively capture spatial-temporal dynamics for improved human action recognition in depth videos as HDDPDI. [21] present and compares two ConvLSTM-based approaches for real-time human action recognition using depth videos, demonstrating that the stateful model achieves superior accuracy and efficiency while preserving privacy.

### 6.1. I2I model performance at schematic threshold configuration

We have conducted statistical analyses to evaluate the reliability of our results adequately. Specifically, each Schematic Threshold Configuration formulates it. These formulated schemes were applied to evaluate the performance metrics and validating the proposed model. The significance levels is validated with the outcome which have been represented in below table.

## 7. Discussion

**Fig 9:**
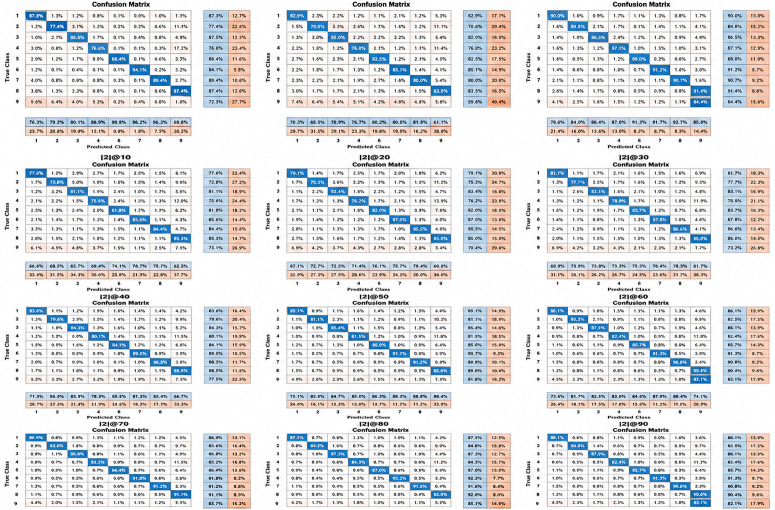
The Confusion matrices reveal the performance of all the variants of Schematic Threshold Configuration. The matrices are consisting of True and Predicted classes for the specific Schematic Threshold Configurations.

Based on the results found for the recognition of action using depth data, the HoD features have been compared for their incorrect information. The effect of neglecting zeros could be easily visualized through experimental analysis described in Fig 9 of Model performance as Confusion Matrices. Incorporating sensitivity analysis of feature thresholds and their impact on precision and F1-score would indeed enhance the robustness and validation of our findings.

Addressing ethical concerns, including the privacy implications of deploying depth cameras in medical settings, is crucial to ensure responsible and secure adoption of such technologies.

While there does exist work regarding deep architectures that combine handcrafted and learned features for HAR, the presented I2I model introduces a unique way of incorporating the HoD representation into a structured, geometry-preserving input to sequential learning layers. Unlike other works relying on depth maps or DDIs as direct inputs, HoD encodes patterns of depth distribution at multiple spatial scales, so that the network can grasp invariant geometric clues prior to temporal modeling. This enables the LSTM layers to capture temporal dependencies across already compact and discriminative geometric descriptors, thus improving both interpretability and reducing computational costs.

Another distinguishing factor of the approach is the proposed threshold-based feature selection mechanism. Other than using generic dimensionality reduction methods, such as PCA or L1 regularization, the thresholding based on the proportion of zero values represents a domain-informed approach custom-suited for the sparsity in depth histograms. This adaptively suppresses those low-activation bins that do not contribute much to action differentiation, thereby refining the feature space with a minimal computational cost. Such targeted reduction is more suitable for depth-based representations where irrelevant or rarely activated patterns can mask meaningful temporal variations.

Apart from ConvLSTM and DDI methods, I2I differs from both in function and behavior: ConvLSTM processes raw pixel-level data, requiring large-scale training and possibly sensitive to viewpoint or noise; while early aggregation of temporal information in DDIs may lead to the loss of fine-grained motion cues. Instead, I2I operates at an intermediate semantic level, filling the gap between handcrafted structural encoding (HoD) with deep temporal modeling (LSTM) for maintaining temporal detail while keeping robustness to spatial variation. Empirically, I2I yields superior or comparable accuracy with fewer parameters, highlighting its efficiency and stability across varying motion complexities.

The novelty of the I2I model lies in its embedding of depth histogram encoding, sparsity-aware feature refinement, and sequence learning for each task, hence forming a compact yet powerful pipeline distinct from other existing depth-based approaches to HAR.

## 8. Conclusion

In conclusion, the proposed model I2I performed proficiently with the Depth data to identify medical-related actions with more precision and accuracy. The proposed model could be a value-added asset for the Robot Vision community that benefits the issues with its vision capabilities. The accuracy of the system has to be substantially enhanced for broaden its applicability in real-world use, particularly for medical disorders that are challenging to diagnose. With this objective in mind, we intend to investigate and study more detailed characteristics associated with medical disorders and enhance depth data with more representative and meaningful features extracted from depth-based data for increased precision. Currently, the proposed model is limited to a single person medical action. The future endeavors could develop the similar for multi person activities also in a domain other than medical. Integrating the I2I approach into assistive robotics and healthcare monitoring systems could significantly enhance their ability to interpret and respond to complex human actions in real-time. This integration would improve system adaptability, enabling more efficient and personalized support for individuals in need. Future work could investigate the integration of depth data with complementary modalities, such as thermal or infrared imaging, to enhance the accuracy and robustness of action recognition systems. Such multimodal approaches would provide richer contextual information, enabling more reliable performance across diverse environments and scenarios.
